# Noise-Parameter Uncertainties: A Monte Carlo Simulation

**DOI:** 10.6028/jres.107.037

**Published:** 2002-10-01

**Authors:** J. Randa

**Affiliations:** National Institute of Standards and Technology, Boulder, CO 80305

**Keywords:** amplifier noise, measurement errors, noise, noise measurement, simulation, uncertainty

## Abstract

This paper reports the formulation and results of a Monte Carlo study of uncertainties in noise-parameter measurements. The simulator permits the computation of the dependence of the uncertainty in the noise parameters on uncertainties in the underlying quantities. Results are obtained for the effect due to uncertainties in the reflection coefficients of the input terminations, the noise temperature of the hot noise source, connector variability, the ambient temperature, and the measurement of the output noise. Representative results are presented for both uncorrelated and correlated uncertainties in the underlying quantities. The simulation program is also used to evaluate two possible enhancements of noise-parameter measurements: the use of a cold noise source as one of the input terminations and the inclusion of a measurement of the “reverse configuration,” in which the noise from the amplifier input is measured directly.

## 1. Introduction

Propagation of uncertainty in measurements of amplifier or device noise parameters can be a complicated task that does not admit an analytical solution. The dependence of the noise parameters on the measured quantities is generally nonlinear, and the noise parameters are typically determined by a least-squares fit to an overdetermined system of equations. Monte Carlo methods are well suited to such problems. They have been used to compare different choices of input terminations [1]–[3] in noise-parameter measurements, and have recently been used to study the dependence on the uncertainties in the underlying quantities [4], [5].

The present paper extends the work of [4] and [5] in several respects. The possibility of correlations among uncertainties in the underlying quantities has been added to the simulator, as has the choice of either a Gaussian or a rectangular distribution for uncertainties in the ambient temperature. The presence of correlations in particular can lead to important effects in the final uncertainties. Also, a different analysis program has been used. The analysis program used in the earlier work lumped together the device under test (DUT) and the receiver used in the measurement. The uncertainties in the noise parameters of the DUT were obtained by assuming that the DUT and the receiver could be disentangled without the introduction of any additional uncertainty. Equivalently, the uncertainties arising from the power measurement were all contained in one power uncertainty, assuming a perfectly matched, noiseless power meter. The present work uses a different analysis program, which includes a full, realistic estimate for the uncertainty in measurement of the output of the DUT for the different input sources. A highly abridged summary of the present work was presented in [6].

We refer to the gain and the noise parameters, which are the quantities to be determined in typical amplifier noise measurements, as the output variables, to distinguish them from what we will call the underlying quantities. The underlying quantities are those that are not themselves the object of the measurement, but that must be known or measured in order to determine the output variables. The underlying variables comprise the noise temperatures and reflection coefficients of the input terminations, the output noise temperature or power from the amplifier for each of the input terminations, and the *S*-parameters [7] of the amplifier (other than |*S*_21_|). The ambient temperature is considered an underlying variable since most of the input terminations are passive devices at ambient temperature. The work reported here uses a simulation program for amplifier noise measurements, along with a companion analysis program, to estimate the uncertainties in the output variables for known values of the underlying variables. The dependence of the uncertainty in each output variable on the most important of the underlying uncertainties is computed, including the effect of correlations among the errors in the underlying quantities. The total uncertainties are given for some representative sets of underlying uncertainties. The simulation program is also used to evaluate two possible enhancements of noise-parameter measurements: the use of a cold noise source as one of the input terminations, and the inclusion of a measurement of the “reverse configuration,” in which the noise from the amplifier input is measured directly.

The following section contains the background theory for the work, both a review of the formalism used to describe amplifier noise parameters and a discussion of the simulation process. Section 3 presents results obtained for the noise-parameter uncertainties and discusses some general features of those results. Section 4 summarizes the work and discusses possible future extensions.

## 2. Theory

### 2.1 Formulation

The formalism used is based on the wave representation of the noise matrix. It is essentially the same formalism as that of [8], but with a few differences in notation. This formulation of noise parameters is convenient because of its versatility: it naturally accommodates measurements in the “reverse” direction, and it provides a simple treatment of isolators. The normalization is such that the spectral power density is given by the square of the absolute value of the wave amplitude. We assume that the noise amplitudes are approximately constant in a small bandwidth (1 Hz, for example) around the frequency of interest, and we have divided out that bandwidth. Throughout this paper, the term “noise temperature” denotes the available noise power spectral density divided by the Boltzmann constant *k*_B_.

**Fig. 1:**
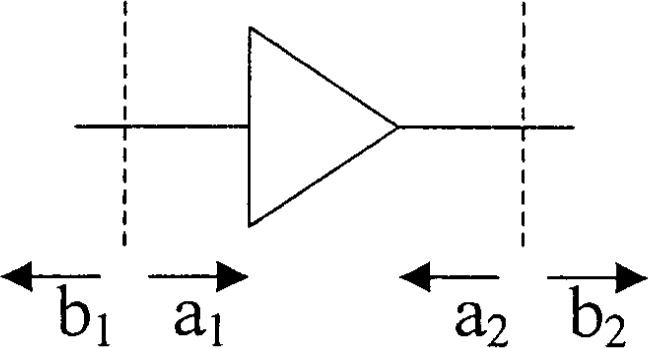
Conventions for Eq. (1).

The amplifier (or transistor) is assumed to be a linear two-port. Its behavior can therefore be represented by

$$\left(\begin{array}{c} b_{1}\\ b_{2} \end{array}\right)=S\left(\begin{array}{c} a_{1}\\ a_{2} \end{array}\right)+\left(\begin{array}{c} {\hat{b}}_{1}\\ {\hat{b}}_{2} \end{array}\right),$$ (1)

where ***S*** is the usual scattering matrix, *a*_1,2_ and *b*_1,2_ are the usual incident and outgoing travelling waves, as in Fig. 1, and ${\hat{b}}_{1}$ and ${\hat{b}}_{2}$ represent the contribution from the intrinsic noise of the amplifier, present even in the absence of any incident wave. The intrinsic noise wave amplitudes ${\hat{b}}_{1}$ and ${\hat{b}}_{2}$ are not themselves measured; rather the measured noise characteristics of the amplifier are the elements of the intrinsic noise matrix

$${\hat{N}}_{ij}\equiv \langle {\hat{b}}_{i}b_{j}^{*}\rangle ,$$ (2)

where the star indicates complex conjugate, and the brackets indicate a time or ensemble average (assumed to be the same). The four independent elements are $\langle {\left|{\hat{b}}_{1}\right|}^{2}\rangle$, $\langle {\left|{\hat{b}}_{2}\right|}^{2}\rangle$, and the real and imaginary parts of $\langle {\hat{b}}_{i}b_{2}^{*}\rangle$. For notational convenience, we define

$$k_{B}X_{1}\equiv \langle {\left|{\hat{b}}_{1}\right|}^{2}\rangle ,k_{B}X_{2}\equiv \langle {\left|{\hat{b}}_{1}/S_{21}\right|}^{2}\rangle ,k_{B}X_{12}\equiv \langle {\hat{b}}_{1}{\left({\hat{b}}_{2}/S_{21}\right)}^{*}\rangle ,$$ (3)

where the *X* parameters have the dimensions of temperature (K). Division of ${\hat{b}}_{2}$ by *S*_21_ has the effect that the *X* parameters are all approximately the same order of magnitude, which is convenient in the data fitting and also in making approximations or arguments about the relative importance of different terms. Although all the calculations for this paper were done in terms of the *X* parameters, the results will be given in terms of the conventional IEEE parameters [9]. The relationship between the two sets of parameters is easily obtained from the relationship between the noise matrix and the IEEE parameters [8]; the equations are given in [5], and we do not reproduce them here. The particular form of the IEEE parameters that we use is defined by

$$T_{e}=T_{\min}+t\frac{{\left|\Gamma_{\text{opt}}-\Gamma_{G}\right|}^{2}}{{\left|1+\Gamma_{\text{opt}}\right|}^{2}\left(1-{\left|\Gamma_{G}\right|}^{2}\right)}$$ (4)

where the four parameters are *T*_min_, *t*, and the complex *Γ*_opt_. *T*_e_ is the effective input noise temperature due to noise from the amplifier itself; *T*_min_ is the minimum value of *T*_e_; *Γ*_opt_ is the value of the input reflection coefficient for which the minimum of *T*_e_ occurs; and *t* controls how rapidly *T*_e_ increases as the input reflection coefficient *Γ*_G_ moves away from *Γ*_opt_.

**Fig. 2:**
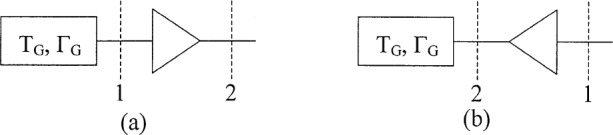
Forward (a) and reverse (b) measurement configurations.

Two measurement configurations will be considered, the forward configuration of Fig. 2(a) and the reverse configuration of Fig. 2(b). The forward configuration is the usual configuration for measuring amplifier noise properties, but the reverse configuration can also be measured [8], [10]–[12], and it provides a very good determination of the parameter *X*_1_. The output noise temperature for the two configurations can be written in terms of the scattering and noise parameters of the amplifier and the reflection coefficient *Γ*_G_ and noise temperature *T*_G_ of the source or generator. For the forward configuration, the equation is

T2=|S21|2(1−|ΓGS|2){(1−|ΓG|2)|1−ΓGS11|2TG+|ΓG1−ΓGS11|2×X1+X2+2Re[ΓGX121−ΓGS11]}, (5)

and for the reverse configuration it takes the form

T1=1(1−|ΓGS′|2){|S12|2(1−|ΓG|2)|1−ΓGS22|2TG+|S12S21ΓG1−ΓGS22|2×X2+X1+2Re[S12S21ΓGX121−ΓGS22]}, (6)

where *Γ*_GS_ is the reflection coefficient of the amplifier and source at plane 2 in Fig. 2(a), and $\Gamma_{\text{GS}}^{\prime }$ is the reflection coefficient of amplifier and source at plane 1 in Fig. 2(b),

$$\begin{array}{l} \Gamma_{\text{GS}}=S_{22}+\frac{\Gamma_{G}S_{21}S_{12}}{\left(1-\Gamma_{G}S_{11}\right)}\\ \Gamma_{\text{GS}}^{\prime }=S_{11}+\frac{\Gamma_{G}S_{12}S_{21}}{\left(1-\Gamma_{G}S_{22}\right)} \end{array}$$ (7)

Equations (5) and (6) are for the noise temperature at the indicated reference plane (1 or 2), since that is what the NIST radiometer measures. Equations for the power delivered to a receiver connected at that reference plane can be obtained simply by introducing a mismatch factor [5].

In Eqs. (5) and (6), the quantities *Γ*_G_, *Γ*_GS_, $\Gamma_{\text{GS}}^{\prime }$, and the *S*-parameters (except |*S*_21_|) can all be accurately measured with a vector network analyzer (VNA); and *T*_G_ is assumed to be known. For the composite reflection coefficients, *Γ*_GS_ and $\Gamma_{\text{GS}}^{\prime }$, the program offers a choice: they can be measured directly or they can be computed by cascading the measured values of *Γ*_G_ and the amplifier’s *S*-parameters. The results presented in this paper all assumed that they were obtained from the cascade computation. The quantities that must be determined from the noise measurements are the noise parameters (*X*_1_, *X*_2_, and *X*_12_) and |*S*_21_|^2^ = *G*_0_. These five parameters are determined using a slight variation on a standard method [13]: the output noise temperature (rather than power as in [13]) is measured for a number of different input noise temperatures, and a least-squares fit is performed to the resulting set of equations (5) and possibly (6). Most of the measurements will be of the forward configuration, but we will also investigate the effect of including a measurement of the reverse configuration. The least-squares fit was weighted by the inverse square of the estimated uncertainty in the measured output temperature. The effect of the weighting is small unless the measurements include one of the reverse configuration, which has an output temperature many orders of magnitude smaller than the forward measurements.

A convenient feature of the *X* parameters is that if a reverse measurement is not present, the equations can easily be put in a linear form,

$$T_{\text{out},i}=\sum_{j=1}^{5}a_{i,j}Z_{j},$$ (8)

with *Z*_1_ = *G*_0_*X*_1_, *Z*_2_ = *G*_0_*X*_2_, *Z*_3_ = *G*_0_ *Re X*_12_, *Z*_4_ = *G*_0_ *Im X*_12_, *Z*_5_ = *G*_0_. In practice, we have both a full nonlinear fitting routine, which can accommodate any combination of measurements, and a linear routine, which can be used if only forward measurements are made. We have checked that the two programs yield identical results in cases where both can be used.

To better understand some of the results that will be obtained below, it is helpful to consider some general features of Eqs. (5) and (6), much in the manner of [14]. If *Γ*_G_ = 0, Eq. (5) reduces to the familiar form for the matched case

$$T_{2}=\frac{G_{0}}{\left(1-{\left|S_{22}\right|}^{2}\right)}\{T_{G}+X_{2}\},$$ (9)

which indicates that *X*_2_ can be identified as *T*_e0_, the effective input noise temperature for the matched case. Equation (9) also demonstrates that two forward measurements with *Γ*_G_ = 0 but with different *T*_G_ would suffice to determine *G*_0_ and *X*_2_. Similarly, one reverse measurement with *Γ*_G_ = 0 would determine *X*_1_. In principle, the real and imaginary parts of *X*_12_ could then be determined by two measurements with |*Γ*_G_| = 1, but with different phase. In practice, of course, perfect terminations are rare, and we also want to include redundant measurements to insure a robust method. Nonetheless, the qualitative features just mentioned persist when we include multiple measurements and the least-squares fit. *G*_0_ and *X*_2_ are determined to large extent by measurements with the hot and ambient matched sources, and *X*_1_ is determined by the reverse measurement—if it is performed. The other measurements serve primarily to determine *X*_12_.

### 2.2 Simulation

A good description of the use of Monte Carlo simulation to estimate uncertainties is given in [15]. For the simulation, we first chose “true” values for the underlying quantities. These comprise the noise and scattering parameters of the amplifier and the noise temperature *T*_G,_*_i_* and reflection coefficient *Γ*_G,_*_i_* of each termination. We then chose uncertainties for the *S_ij_*, *T*_G,_*_i_*, *Γ*_G,_*_i_*, and the measurements of the output noise temperature. We also chose a value for the connector variability. All measurement distributions were taken to be Gaussian except for the ambient temperature. All the results in this paper used a rectangular distribution for the ambient temperature, to simulate the effect of a laboratory thermostat, but the program allows a choice of either rectangular or Gaussian distribution for the ambient temperature.

**Fig. 3:**
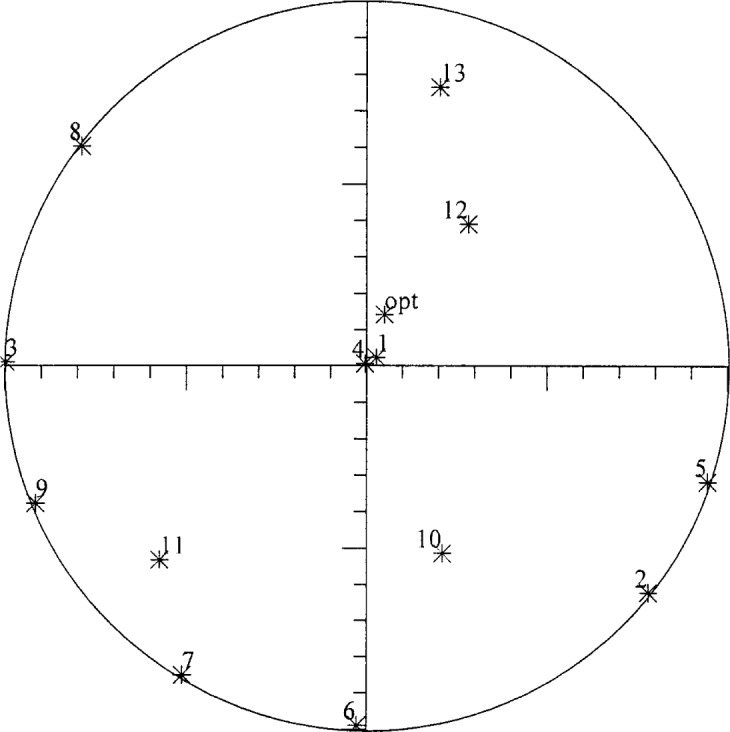
Distribution of input reflection coefficients in and on the unit circle in the complex plane. The location of *Γ*_opt_ is marked by “opt”.

In studies of noise-parameter measurements, there are a myriad of variables whose interdependent effects can be studied. The current paper focuses on the dependence of the noise-parameter and gain uncertainties on the uncertainties in the underlying quantities, for both correlated and uncorrelated uncertainties. For the other variables entering the problem, typical or representative values are chosen. Thus, for the set of input terminations we chose 13 terminations, one of them hot, the rest at ambient temperature, with reflection coefficients distributed in the complex plane as shown in Fig. 3, where point 1 is the hot termination. Similarly, we are not studying the manner in which the uncertainties depend on the actual noise parameters themselves, so we consider just one particular set of noise parameters, measured for a low-noise amplifier at a single frequency. The values used for the “true” values were *G*_0_ = 2399 (33.80 dB), *X*_1_ = 43.402 K, *X*_2_ = 113.1509 K, *X*_12_ = (−5.8228 + 8.4897*j*) K, corresponding to IEEE parameters *T*_min_ = 109.6 K (*F*_min_ = 1.392 dB), *Γ*_opt_ = 0.050 + 0.142 j, and *t* = 176.3 K.

We generated simulated measured values for the *S_ij_*, *T*_G,_*_i_*, and *Γ*_G,_*_i_* in the standard manner, randomly choosing a value from the appropriate distribution centered at the true value. For the complex quantities, real and imaginary parts were generated independently. To generate the simulated noise-temperature measurement, we first calculated the true output noise temperature from the equation for output temperature, using the true values for the noise parameters and the termination noise temperatures and using values for the *S*-parameters and the reflection coefficient that differed from the true values by random deviates chosen from the connector variability distribution. This complication was included to account for the fact that the “true” value for a reflection coefficient or *S*-parameter varies with each connection. Once the true output temperature for the given connection was calculated, a simulated measured value for it was generated using the uncertainty in the noise-temperature measurement as the standard deviation. A complete simulated measurement set then consisted of the measured values for *S_ij_* and the measured *T*_G,_*_i_*, *Γ*_G,_*_i_*, and *T*_out,_*_i_* for each of the 13 terminations.

The complete simulated measurement set was analyzed and the noise parameters and gain determined in the same way as for a real data set. A weighted-least-squares fitting routine was used. To assess the uncertainties in the noise parameters, we generated a large number *N*_sim_ of simulated measurement sets with the given uncertainties in the underlying quantities. Each simulated measurement set was analyzed to produce a set of “measured” noise parameters, yielding *N*_sim_ measured values for each parameter. The average and standard deviation of the measured values were computed. The uncertainty in a single measurement of a parameter was then computed by combining the standard deviation in quadrature with the difference between the average and the true value. This is just the root-mean-square error (*RMSE*) of the sample,

$$u(y)\approx RMSE(y)=\sqrt{Var(y)+{\left(\bar{y}-y_{\text{true}}\right)}^{2}}.$$ (10)

Statistics for *Γ*_opt_ were computed for its real and imaginary parts, not its magnitude and phase. For all the results in this paper, *N*_sim_ = 1000 was used.

Correlations in the underlying uncertainties were introduced by having separate uncertainties for correlated and uncorrelated errors. For example, two uncertainties were associated with the ambient temperature, one for uncorrelated errors $\left(\sigma_{T_{a},\text{unc}}\right)$ and the other for correlated errors $\left(\sigma_{T_{a},\text{cor}}\right)$. When generating the measured value for an ambient-temperature input termination, we added two random deviates to the true value, an uncorrelated component with $\left(\sigma_{T_{a},\text{unc}}\right)$ that is different for each termination and a correlated component with $\left(\sigma_{T_{a},\text{cor}}\right)$ that is the same for each. A similar procedure was followed for the measured output temperatures; there was a correlated error common to all the measurements and an uncorrelated error that is different for each. There was some question whether the error in the noise temperature of the hot input termination should be correlated with the errors in the measurement of the output noise temperatures, since often the same hot noise source that is used as an input termination is also used to calibrate the radiometer (or noise figure meter). The simulation program allows it to be either correlated or not, and all the results in this paper assumed that the hot input noise temperature was correlated with the measurements of the output noise temperatures. For the reflection coefficients, which are complex, real and imaginary parts were treated separately. Correlations were allowed among all the real parts and all the imaginary parts, but not between real and imaginary parts. This choice was a natural extension of the treatment in NIST’s uncertainty analysis of noise-temperature measurements, but in future work we intend to allow input of magnitude and phase uncertainties, which is the more common practice.

### 2.3 Program Verification

The program was checked in several ways to bolster confidence in the results. The fitting and analysis modules were tested separately and in tandem. Both the linear and the nonlinear fitting routines were part of a popular commercial package, but we ran tests nonetheless to verify that they were being used properly. The fitting module was run with a set of correct values for all the *T*_out_’s to verify that it found the correct solution in that case. We also ran the module with a range of different starting points and verified that it always found the same solution, and we manually verified that the solution was a minimum of the fitting function. Finally, as mentioned above, we used both a linear and a full nonlinear routine for a test case with only forward measurements and verified that they produced the same result. The simulator was checked by analyzing a set of output data and verifying that the data sets for all parameters had the correct mean, standard deviation, and correlations. We also visually inspected graphs of the output data to verify that there were no surprises. For the full Monte Carlo program, combining the simulator and the analysis routine, we compared results for different numbers (100, 1000, 10 000) of simulated measurement sets to verify that *N*_sim_ = 1000 was sufficient. We also compared results using different seed values for the random-number generator, thus generating and analyzing different data sets. The results of these two exercises indicated that the resulting uncertainties were stable to within two or three percent. We ran the program with all underlying uncertainties set to zero and verified that the resulting noise parameters were equal to the true values for all 1000 simulated measurement sets. We also used a spreadsheet program to check that the statistics of the simulated measurement results were being computed correctly by the program. It is, of course, still possible that an undetected error lurks somewhere in the program, but at this point it appears unlikely.

## 3. Results

Three different types of results will be presented. The first and largest set of results will demonstrate the dependence of the uncertainties in the output parameters on the uncertainties in the underlying parameters. The second set of results is for a selection of typical cases, meant to be representative of the uncertainties achievable in some common scenarios, and the third set of results is an investigation of the effect of two possible enhancements of the measurement set.

**Fig. 4:**
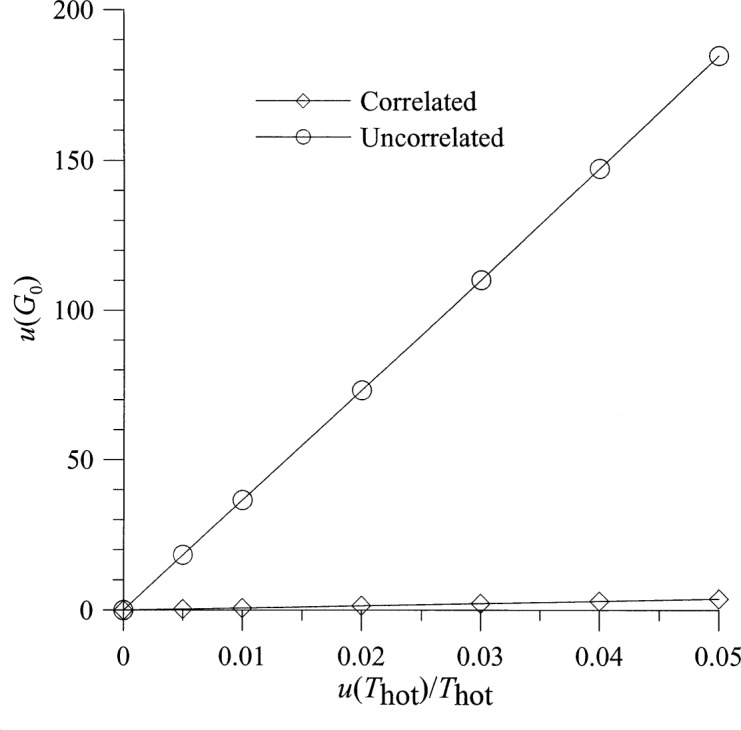
Dependence of the uncertainty in the gain on the fractional uncertainty in measurement of hot noise temperatures, for correlated and uncorrelated uncertainties in the hot noise-temperature measurements.

**Fig. 5:**
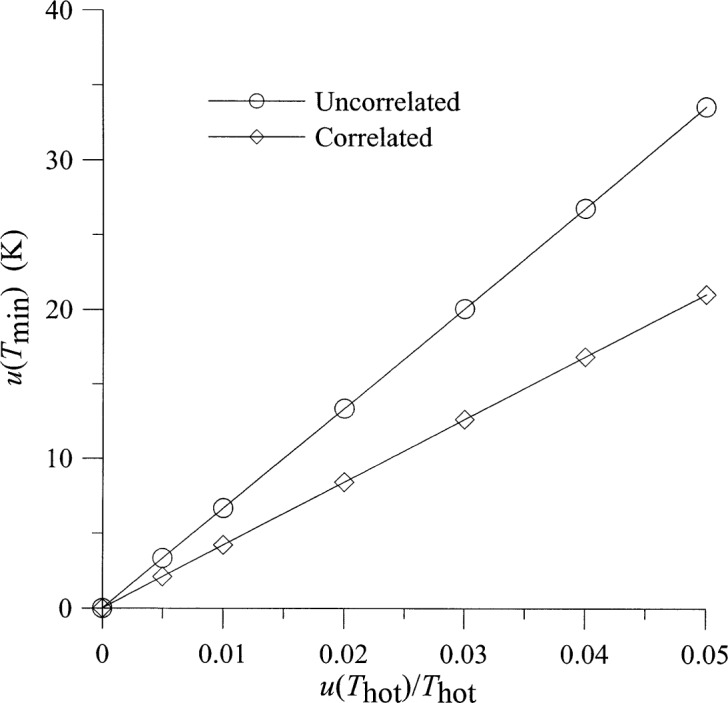
Dependence of the uncertainty in *T*_min_ on the fractional uncertainty in measurements of hot noise temperatures.

**Fig. 6:**
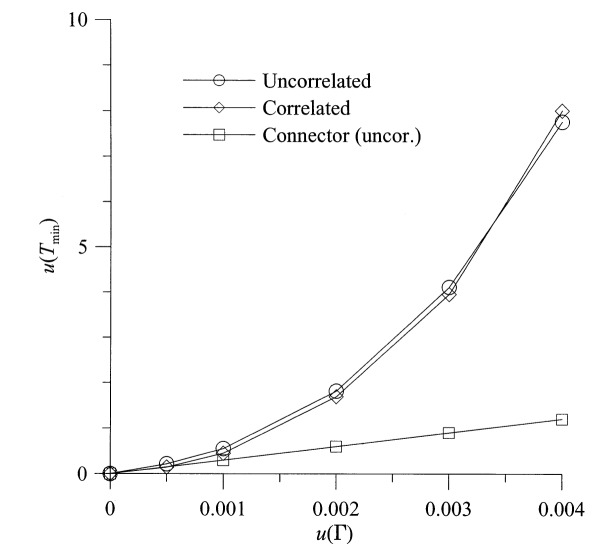
Dependence of the uncertainty in *T*_min_ on the uncertainty in the reflection coefficients of the input terminations and the connector variability.

**Fig. 7:**
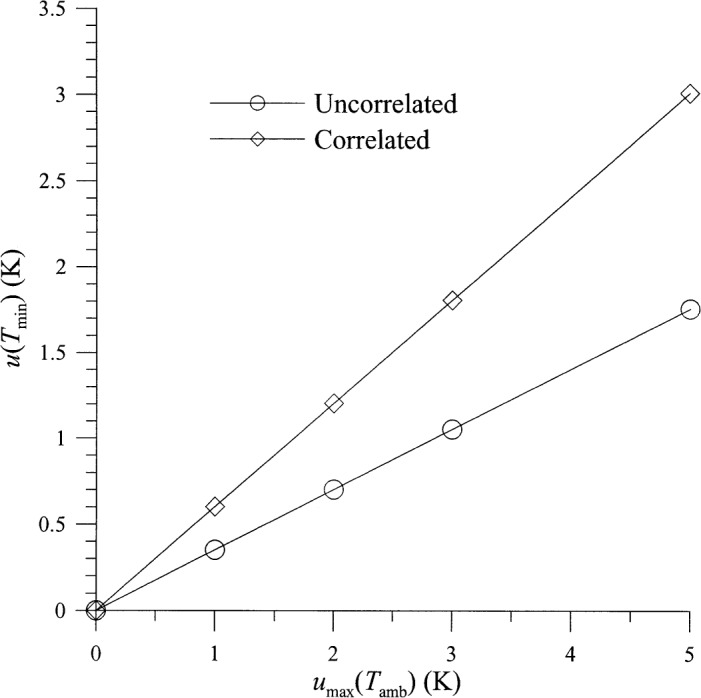
Dependence of the uncertainty in *T*_min_ on the uncertainty in the temperature of the ambient input terminations.

**Fig. 8:**
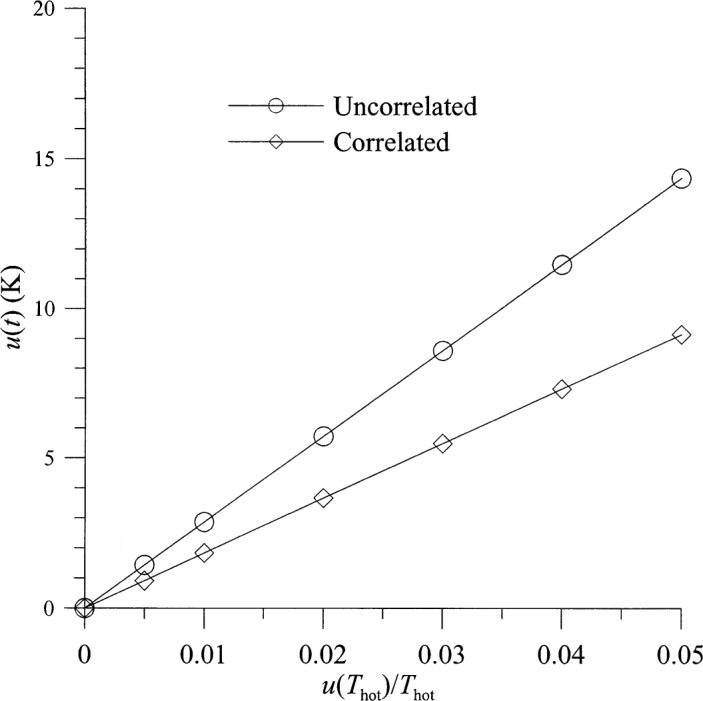
Dependence of the uncertainty in *t* on the fractional uncertainty in measurements of hot noise temperatures.

**Fig. 9:**
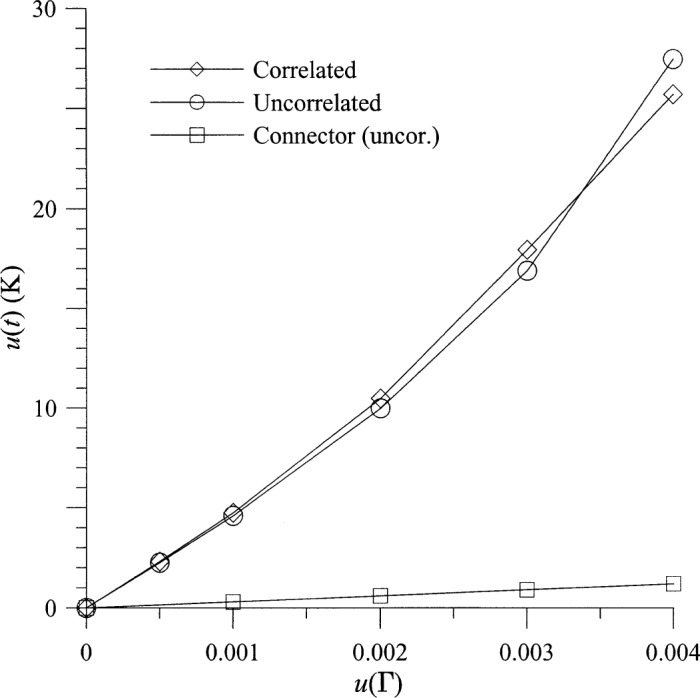
Dependence of the uncertainty in *t* on the uncertainty in the reflection coefficients of the input terminations and the connector variability.

**Fig. 10:**
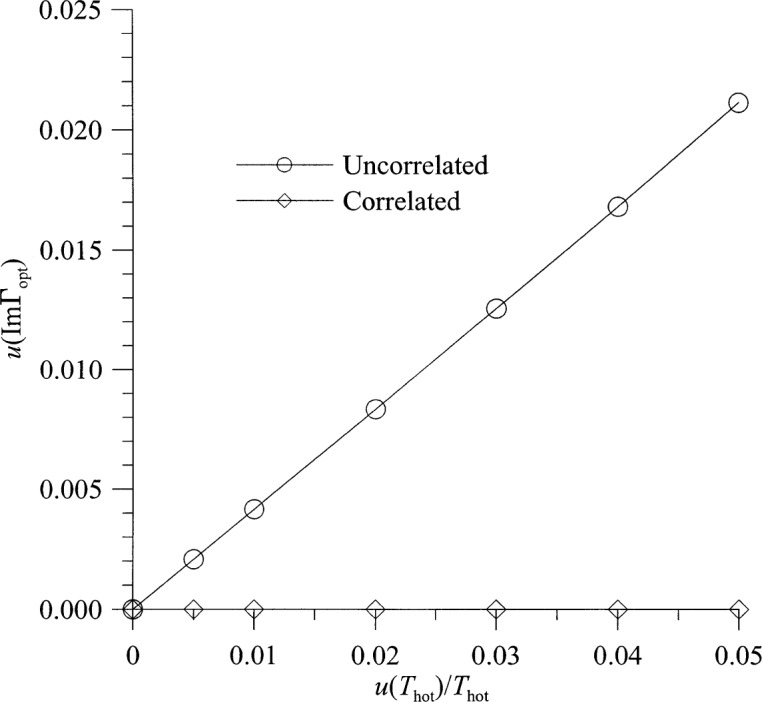
Dependence of the uncertainty in *ImΓ*_opt_ on the fractional uncertainty in measurements of hot noise temperatures.

**Fig. 11:**
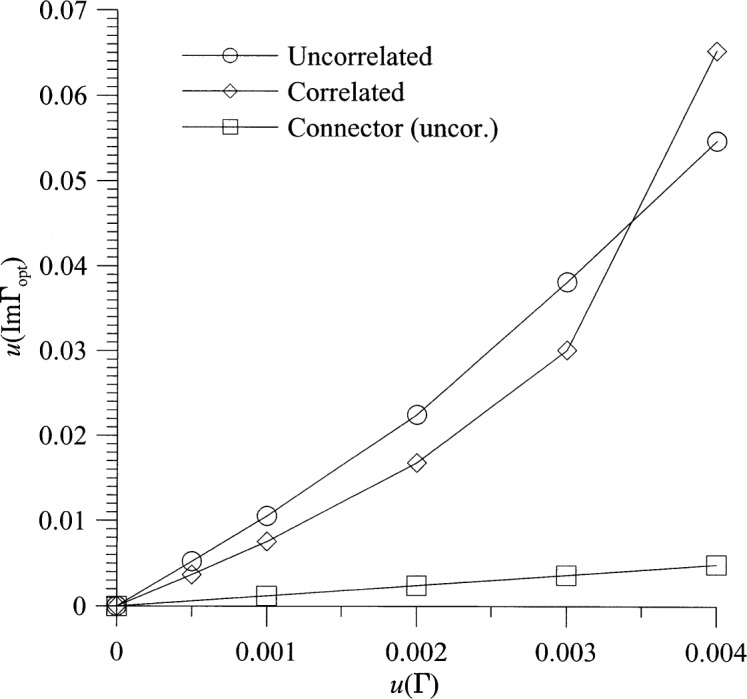
Dependence of the uncertainty in *ImΓ*_opt_ on the uncertainty in the reflection coefficients of the input terminations.

Selected results for the dependence of the output uncertainties on the underlying uncertainties are shown in Figs. 4–11. To isolate the effect of a single underlying uncertainty, these figures show the dependence on one underlying uncertainty, with all other underlying uncertainties set to zero. Figure 4 shows the dependence of the uncertainty in the (reduced) gain on the fractional uncertainty in the measurement of hot noise temperatures for both the case with the errors in all hot noise temperatures completely uncorrelated, and the case with the errors in the hot noise temperatures perfectly correlated. The fractional uncertainty in the hot noise temperature applies both to the hot source used as one of the input terminations and to the measurements of the output noise temperatures. Figure 4 indicates that the uncertainty in measuring the noise temperature has a major effect on the uncertainty in the gain, as would be expected. What may be rather surprising is that if the uncertainties in the noise-temperature measurements are all perfectly correlated, the resulting uncertainty in the gain is very small. This can be understood by recalling from Eq. (9) that the gain is determined primarily by the difference in two noise-temperature measurements, and correlated errors cancel in taking the difference. A similar, but less pronounced, effect occurs for the uncertainty in *T*_min_, Fig. 5. For those accustomed to measuring the characteristics in decibels, an uncertainty in *G*_0_ of 100 (for *G*_0_ = 2400) corresponds to about 0.18 dB, and an uncertainty of 20 K in *T*_min_ (for *T*_min_ = 110 K) corresponds to an uncertainty of approximately 0.2 dB in the minimum noise figure.

The uncertainty in the noise temperature of the hot source and in the measurement of the output temperature is the most important contribution to the uncertainties in *G*_0_ and *T*_min_. The uncertainty in the input reflection coefficients has very little effect on the uncertainty in *G*_0_, but it does contribute to the uncertainty in *T*_min_, as shown in Fig. 6. Uncertainties in the real and imaginary parts of the input reflection coefficients were taken to be equal and uncorrelated and are both called *u*(*Γ*). Figure 6 also shows the small effect of the connector variability on the uncertainty in *T*_min_. The uncertainty in the ambient temperature *T*_amb_ has very little effect on any of the measured parameters (although it may, of course, affect the actual properties of the device itself). Its most significant effect is on the uncertainty in *T*_min_, which is shown in Fig. 7. Since a rectangular distribution was used, the maximum value of the error in *T*_amb_ was used as the abscissa.

Figures 8 and 9 show the uncertainty in *t* as a function of the fractional uncertainty in *T*_hot_ and in the reflection coefficients and connector variability. The uncertainty in the imaginary part of *Γ*_opt_ is shown in Fig. 10 as a function of the uncertainty in *T*_hot_, and in Fig. 11 as a function of the uncertainty in the real or imaginary part of the reflection coefficients of the input terminations. The uncertainty in the real part of *Γ*_opt_ exhibits qualitatively similar behavior. For both *t* and *Γ*_opt_, the effect of the uncertainty in *T*_amb_ is negligible.

The results thus far demonstrate the dependence of the noise-parameter uncertainties on individual underlying uncertainties, but they do not tell the total uncertainty due to the combined effect of all the underlying uncertainties. For that we evaluated the uncertainties in the noise parameters and gain resulting from a few sets of underlying uncertainties that we consider typical or representative of common situations. The three cases are labeled Average (meant to represent average industrial laboratory measurements), Good (representing measurements by a very good industrial laboratory or a good standards laboratory), and Very Good (meant to represent national standards laboratories). The underlying uncertainties for the different cases are given in Table 1, and the resulting uncertainties in the noise parameters and gains are tabulated in Table 2. In Table 1, *u*_Th,frac_ is the fractional uncertainty in the noise temperature of the hot input source, *u*_Ta_ is the uncertainty in the ambient temperature, *u*(*Γ*) is the uncertainty in the real and imaginary parts of the reflection coefficients, and *u*_con_ is the uncertainty due to connector variability. *F*_min_ in Table 2 is the minimum noise figure in decibels, defined as

$$F_{\min}=10\;\log\left(\frac{T_{\min}+T_{0}}{T_{0}}\right),$$ (11)

where *T*_0_ = 290 K. The results of Table 2 require little explanation. Because the uncertainty in most of the noise parameters is dominated by the contribution of a single underlying uncertainty, good approximations to most of the results of Table 2 could be read from Figs. 4–11.

The Monte Carlo simulation can also be used to compare different measurement strategies. Two variations were considered. One was the inclusion of a cold input noise source, either instead of, or in addition to, the hot noise source. Equation (8) indicates that *X*_2_ = *T*_e0_ occurs in conjunction with *T*_G_; if *X*_2_ is small, as it is for a low-noise amplifier, it should be more easily determined if *T*_G_ is also small, since the *fractional* uncertainty in *T*_G_ is typically about the same for hot or cold noise sources. The second variation is to include a measurement of the reverse configuration, with a matched, ambient termination on the output of the amplifier [8], [10]–[12] as in Fig. 2(b) and Eq. (6). This gives a good, direct measurement of the parameter *X*_1_, and one would therefore expect it to improve the determination of the noise parameters, perhaps quite significantly.

Table 3 gives the results for the uncertainties in the noise parameters and gain when these alternative measurement strategies are implemented. As a baseline, we use the VG results from above, which are labeled VG-h here, since they used a hot input noise source as the nonambient source and no measurement of the reverse configuration. For VG-c, we replaced the hot source by a cold source (*T* = 78 K), keeping everything else the same, and VG-hc uses both hot and cold sources. VG-hr indicates that a measurement of the reverse configuration was added to the VG-h case. Similarly, VG-cr indicates the addition of a reverse measurement to VG-c, and VG-hcr adds a reverse measurement to VG-hc. In all cases, the same 12 ambient-termination, forward-configuration measurements were also made.

The results are not entirely as expected. If we first consider the cold input source, we see that using a cold, rather than a hot, input source improves the uncertainty in *T*_min_ and *t*, but it increases the uncertainty in *G*_0_. Using both a hot and a cold input noise source decreases the uncertainty in all three. The improvement in the determination of *G*_0_ and *t* is small, but the uncertainty in *T*_min_ is reduced by a factor of two from the VG-h case, which used only the hot source, as is the common procedure. This result differs somewhat from the result of [5], which found that substituting a cold source for the hot source decreased all uncertainties. The difference is due to different values for the uncertainty in measuring the output noise, as explained in the appendix.

The results of including a measurement of the reverse configuration are even more surprising. If a reverse measurement with a matched, ambient load is added to our basic set of thirteen forward measurements, the uncertainty in *X*_1_ (not shown in the table) does indeed decrease, as expected, but it is more than offset by an increase in the uncertainty in *X*_2_, and as a result the uncertainty in both *T*_min_ and *t* is increased. The same is true when a reverse measurement is added to VG-c or VG-ch. This result seems counter-intuitive (at least to the author), but it can be understood by noting that a good measurement of *X*_1_ effectively reduces the number of degrees of freedom left in the model, and this reduced number of free parameters must account for all the variation induced by the underlying uncertainties. We checked the result by considering the case in which the true value of *X*_1_ was known *a priori*, and only the remaining three noise parameters and the gain were determined by the fit to the measurements. This case also resulted in increased uncertainties for *T*_min_ and *t* compared to the case in which *X*_1_ was not known and was determined with the other parameters from the fit to the measurement results. We therefore conclude that unless one’s interest is in the value of *X*_1_, it is not always helpful to include a measurement of the reverse configuration. (This conclusion could be very dependent on the values of the noise parameters of the amplifier or device.)

## 4. Summary and Discussion

A Monte Carlo simulation was used to evaluate the uncertainties in noise-parameter measurements arising from uncertainties in the underlying variables, which could be either correlated or not. The dependence of the individual noise-parameter uncertainties on the different underlying uncertainties was shown, and some general qualitative features emerged. The uncertainty in the gain is due almost entirely to the uncertainties in the hot input source and the measurement of the output noise temperature. The uncertainty in *T*_min_ is due primarily to the uncertainty in the hot noise temperature and the measurement of the output noise temperatures, but it also can receive a significant contribution from the uncertainty in the reflection coefficient of the input terminations. Both the reflection coefficients and the hot input and output noise temperatures contribute to the uncertainty in *t*. For *Γ*_opt_ the uncertainties in the input reflection coefficients produce the largest effect, but the uncertainty in measuring the output noise temperature also contributes. The connector variability and the uncertainty in the ambient temperature have little effect on the uncertainties in any of the output variables, except possibly in extreme cases. Changes in the ambient temperature can affect the actual properties of an amplifier, however. Correlations among the underlying uncertainties increase the output uncertainties in some cases and decrease them in others; the most dramatic effects of correlations are reductions in the uncertainty in the gain for correlated errors in measuring the output noise temperature.

The Monte Carlo program was also used to compute the total uncertainties in the output variables for some representative cases and to evaluate two possibilities for improving the accuracy of noise-parameter measurements. We found that inclusion of a cold input noise source, in addition to the hot source usually used, reduced the uncertainty in *T*_min_ by a factor of two. It also reduced the uncertainty in the gain slightly and provided a more robust measurement of the gain. On the other hand, addition of a measurement of the reverse configuration was found to increase the uncertainties in the usual IEEE noise parameters.

There are a few limitations to the present work which remain to be addressed in the future. The results presented were for only one particular set of values of the output parameters. We have evaluated the uncertainties for some other values, but do not yet have general quantitative rules for the output uncertainties. The qualitative conclusions concerning which underlying uncertainties control which output uncertainties are likely to hold in general. One factor that may affect the size of some of the uncertainties is the location of *Γ*_opt_, particularly since there are empty areas in our distribution of input reflection coefficients, Fig. 3. We expect to use the program to perform additional studies of the uncertainties in different situations and of other possible measurement strategies.

There are two modifications that should and will be made to the program itself. The input will be modified so that it accepts uncertainties for the magnitude and phase of the reflection coefficients, rather than for the real and imaginary parts. Also, the program will be modified so that it can accommodate measurement and analysis of the output noise power, rather than noise temperature. Another possibility would be to produce a version that would work with other popular analysis programs. Finally, if there is sufficient interest, the program will be made more user-friendly and made available for distribution.

## Figures and Tables

**Table 1:** Underlying uncertainties used in representative cases

Case	*u*_Th,frac_	$u_{T_{a}}(K)$	*u*(*Γ*)	*u*_con_
Average	0.020	1.0	0.004	0.002
Good	0.010	0.8	0.003	0.001
VG	0.005	0.5	0.002	0.001

**Table 2:** Noise-Parameter uncertainties for representative cases

Case	$U_{G_{0}}(\text{dB})$	$u_{T_{\min}}(K)$	$u_{F_{\text{min}}}\left(\text{dB}\right)$	*u_t_* (K)	$u_{Re\Gamma_{\text{opt}}}$	$u_{Im\Gamma_{\text{opt}}}$
Average	0.13	17.1	0.19	26.1	0.040	0.056
Good	0.07	8.8	0.10	16.9	0.026	0.034
VG-h	0.03	4.2	0.05	9.9	0.016	0.020

**Table 3:** Noise-Parameter uncertainties for alternative strategies

Case	$U_{G_{0}}(\text{dB})$	$u_{T_{\min}}(K)$	$u_{F_{\text{min}}}\left(\text{dB}\right)$	*u_t_* (K)	$u_{Re\Gamma_{\text{opt}}}$	$u_{Im\Gamma_{\text{opt}}}$
VG-h	0.032	4.23	0.05	9.92	0.016	0.020
VG-c	0.051	2.96	0.03	8.85	0.016	0.020
VG-hc	0.026	1.95	0.02	9.71	0.016	0.021
VG-hr	0.040	6.81	0.08	10.94	0.017	0.020
VG-cr	0.066	7.25	0.08	11.71	0.017	0.020
VG-hcr	0.038	6.31	0.07	10.94	0.017	0.020

## 5. Appendix

There is a qualitative difference between the present results and those of [5] when a cold source is substituted for the hot source as the non-ambient input noise source. In [5] the uncertainty in both *G*_0_ and *T*_min_ decreased, whereas in the present work the uncertainty in *G*_0_ increased. The difference is due to the different values assigned to the fractional uncertainty *α* in measuring the output noise, taken to be 0.001 in [5] and 0.01 in the present work. This can be understood by considering the simple, matched case of Eq. (9), further simplified by taking *S*_22_ = 0. Then the two measurements to determine *G*_0_ and *X*_2_ = *T*_e0_ (which controls *T*_min_) are the familiar

$$p_{h}=G_{0}(T_{h}+X_{2}),\;p_{c}=G_{0}(T_{c}+X_{2}),$$ (A.1)

which yield

$$X_{2}=\frac{T_{h}-YT_{c}}{Y-1},\;G_{0}=\frac{p_{h}-p_{c}}{T_{h}-T_{c}},\;Y\equiv \frac{p_{h}}{p_{c}}.$$ (A.2)

We have neglected factors of *k*_B_ and have considered the case in which output power (*p*_h_ and *p*_c_ for hot and cold input) is measured; measurement of output noise temperature yields the same results. If we assume all correlations are absent, then the uncertainty in *X*_2_ can be written as

$$u_{X_{2}}^{2}=\frac{u_{T_{h}}^{2}+Y^{2}u_{T_{c}}^{2}}{{\left(Y-1\right)}^{2}}+{\left(\frac{T_{c}+X_{2}}{Y-1}\right)}^{2}2\alpha^{2},$$ (A.3)

where *α* is the fractional uncertainty in measuring the output power. There are two sources of uncertainty for *X*_2_: how well the input noise temperatures are known $\left(u_{T_{h}},u_{T_{c}}\right)$ and how well the output noise can be measured (*α*). For practical cases with hot (10 000 K ± 100 K) and ambient (296 K ± 0.1 K) input sources and a low-noise amplifier (*X*_2_ ≈ 100 K), the first term dominates for reasonable values of *α*, and $u_{X_{2}}\approx u_{T_{h}}/Y$. If an ambient source and a cold source (80 K ± 0.8 K) are used, the first term of Eq. (A.3) is significantly reduced, and although the second term increases, the net effect is a reduction of $u_{X_{2}}$ for both *α* = 0.01 and 0.001. This is consistent with the present results and those of [5].

The fractional uncertainty in the gain can be written as

$${\left(\frac{u_{G_{0}}}{G_{0}}\right)}^{2}=\frac{\alpha^{2}\left[{\left(T_{h}+X_{2}\right)}^{2}+{\left(T_{c}+X_{2}\right)}^{2}\right]}{{\left(T_{h}-T_{c}\right)}^{2}}+\frac{u_{T_{h}}^{2}+u_{T_{c}}^{2}}{{\left(T_{h}-T_{c}\right)}^{2}}$$ (A.4)

For hot plus ambient-temperature input sources, this is approximately

$${\left(\frac{u_{G_{0}}}{G_{0}}\right)}^{2}\approx \alpha^{2}+{\left(\frac{u_{T_{h}}}{T_{h}}\right)}^{2},$$ (A.5)

whereas for ambient plus cold input sources it becomes

$${\left(\frac{u_{G_{0}}}{G_{0}}\right)}^{2}\approx 2\alpha^{2}+{\left(\frac{u_{T_{c}}}{T_{a}-T_{c}}\right)}^{2}.$$ (A.6)

The situation now is quite different, depending on whether *α* = 0.01 or 0.001. If *α* = 0.001, as in [5], the second term dominates, and the uncertainty is smaller for hot plus ambient than for ambient plus cold. If *α* = 0.01, however, the two terms are comparable, and the uncertainty in the gain is somewhat larger for the cold plus ambient case, as observed in the present work.
