# Erratum: Noise-Parameter Uncertainties: A Monte Carlo Simulation

**DOI:** 10.6028/jres.111.036

**Published:** 2006-12-01

**Authors:** J. Randa

**Affiliations:** National Institute of Standards and Technology, Boulder, CO 80305

[J. Res. Natl. Inst. Stand. Technol., Volume 107, Number 5, September-October 2002, p. 433]

**Erratum:** In Eq. (6), the *X*_12_ in the final term should instead be *X*_12_^*^. Thus, the correct form of Eq. (6) is

T1=1(1−|ΓGS′|2){|S12|2(1−|ΓG|2)|1−ΓGS22|2TG+|S12S21ΓG1−ΓGS22|2X2+X1+2Re[S12S21ΓGX12*1−ΓGS22]}

Results in the paper are unchanged. I am grateful to Tom McKay of RF Micro Devices and Larry Wagner of IBM for calling this to my attention.

